# Measurement of the ^231^Pa/^235^U ratio for the age determination of uranium materials

**DOI:** 10.1007/s10967-018-6247-9

**Published:** 2018-11-11

**Authors:** Zsolt Varga, Adrian Nicholl, Erich Hrnecek, Maria Wallenius, Klaus Mayer

**Affiliations:** European Commission, Joint Research Centre, Directorate for Nuclear Safety and Security, Postfach 2340, 76125 Karlsruhe, Germany

**Keywords:** Uranium, Age determination, Protactinium measurement, Nuclear safeguards, Nuclear forensics

## Abstract

The paper describes the age (production date) determination of uranium reference materials using the ^231^Pa/^235^U ratio. Direct addition of ^237^Np in secular equilibrium with its ^233^Pa daughter was chosen instead of the regular milking of ^237^Np to avoid possible loss of Pa. Sample preparation consists of a fast, one-step procedure. The developed method using ICP-MS for the measurement of ^231^Pa is more precise than alpha spectrometry and is applicable for freshly produced low-enriched uranium materials. The measured ages are in good agreement with the reported production dates, thus the ^231^Pa/^235^U chronometer can be applied for validation of ^230^Th/^234^U in nuclear forensics and safeguards.

**Electronic supplementary material:**

The online version of this article (10.1007/s10967-018-6247-9) contains supplementary material, which is available to authorized users.

## Introduction

Nuclear materials are strictly controlled by the nuclear safeguards regimes. If such material, however, gets out of the regulatory control and is confiscated afterwards, a detailed examination should be performed to identify the intended use, origin and last legal owner of the material [1, 2]. Nuclear forensic analysis uses several signatures, like U or Pu isotopic composition, fuel pellet dimensions, chemical form and impurities, isotope ratios of minor constituents such as S, Sr, Nd, and Pb, to provide hints on the production history of the material and to narrow down the possible facilities being in connection with the material [1–4]. One of the nuclear forensic signatures is the time elapsed since the last chemical or physical purification of the material, commonly called the *age* of the material, can be measured for radioactive, and thus also for nuclear materials [1, 5–7]. This unique opportunity is based on exploiting the presence and decay of radionuclides: when radioactive material is chemically or physically purified from the impurities, also the radioactive decay products are separated. After this separation, the radioactive progenies start to grow-in into the material. By measuring the daughter-to-parent ratio in the sample, the time elapsed since the last separation can be calculated according to the decay equations (Bateman-equations), when assuming that the parent-daughter separation was complete during the process. In contrast to most other nuclear forensic signatures, the production date of the material is a predictive signature, thus it does not require databases or comparison samples for interpretation (i.e. it is a self-explaining signature). This feature makes the age of the material one of the most prominent signatures for attribution.

The age can be calculated as follows:

1 $$t = \frac{1}{{\lambda_{\text{parent}} - \lambda_{\text{daughter}} }}\ln \left( {1 - \frac{{N_{\text{daughter}} }}{{N_{\text{parent}} }} \cdot \frac{{\lambda_{\text{daughter}} - \lambda_{\text{parent}} }}{{\lambda_{\text{parent}} }}} \right)$$

where *λ*_daughter_ and *λ*_parent_ are the decay constants of daughter and parent nuclides, respectively, *N*_daughter_/*N*_parent_ is the amount ratio of the daughter and parent nuclides in the sample, and *t* is the elapsed time since the separation of the radionuclides. The daughter-to-parent ratio (*N*_daughter_/*N*_parent_) is often referred to as *chronometer*, while the elapsed time (*t*) is called the (model) age of the material. Usually ^230^Th/^234^U chronometer is used for age dating of uranium materials. The reason for this is the high chemical dissimilarity between Th and U and the easier measurement of the ^230^Th in trace level. A major drawback for the Pa measurements is the lack of long-lived Pa isotopes (beside the analyte ^231^Pa), which can be used as a spike. The often used short-lived ^233^Pa spike (*T*_1/2_ = 26.98 days) is typically milked from ^237^Np and calibrated against a rock standard (e.g. Table Mountain Latite [8] or measured with gamma spectrometry at fixed geometry to determine the ^233^Pa concentration [9]. Milking of ^233^Pa from the parent ^237^Np is tedious and time consuming, and it cannot be performed before enough ^233^Pa is produced from the decay of ^237^Np, which can take weeks to reach the secular equilibrium. As Pa is prone to adsorption, loss of the ^233^Pa spike cannot be excluded. Another option for milking is to use gamma spectrometry before and after the separation to measure the recovery of ^233^Pa [10]. The ^231^Pa analysis can be carried out by alpha spectrometry in a fixed geometry [10] or inductively coupled plasma mass spectrometry (ICP-MS) [8]. It has to be noted that as ^231^Pa and ^233^Pa decay with a different mode (alpha and beta decay), thus they cannot be measured simultaneously in a quantitative way by radiometric methods. Hence, mass spectrometry is a viable alternative. With mass spectrometry, the measurement of the short-lived ^233^Pa is cumbersome, as measurable amount (i.e. relative high activity) is needed for the precise measurement.

The aim of the present study was to develop a simple, but precise and accurate method for the sample preparation and subsequently precise determination of the ^231^Pa/^235^U ratio in uranium matrices. In order to minimise the possible loss of Pa during milking and evade it e.g. at the end of the measurement by gamma spectrometry, the ^237^Np solution in secular equilibrium with its daughter ^233^Pa was added directly to the sample solution prior to the chemical separation. As more standards are available for ^237^Np, its measurement is much straightforward than preparing a ^233^Pa spike solution. The direct spiking does not require the regular milking of ^233^Pa after the ingrowth from ^237^Np, so the Pa spike is readily and continuously available. The Pa/Np fractionation was checked with gamma spectrometry. The developed method was applied for four U certified reference materials (CRMs) either with certified (model) age determined from the ^230^Th/^234^U ratio or with known production history.

## Experimental

### Reagents and materials

All labware was thoroughly cleaned before use. Suprapur grade hydrochloric, hydrofluoric and nitric acids (Merck, Darmstadt, Germany) were used for the sample preparation. HNO_3_ was further purified by sub-boiling distillation (AHF Analysentechnik AG, Germany). For dilutions ultrapure water was used (Elga LabWater, Celle, Germany). A ^233^U isotopic reference material was used to spike the samples for the uranium concentration measurements by isotope dilution mass spectrometry. The ^233^U concentration in the spike was calibrated against EC NRM 101 uranium metal by thermal ionization mass spectrometry (TIMS). Uranium CRM U-010 standard reference material (nominally 1% ^235^U) from National Bureau of Standards (USA) was used to correct for instrumental mass discrimination of the ICP-MS. Isotopic reference material IRMM-185 (certified *n*(^235^U)/*n*(^238^U) value is (2.00552 ± 0.00060) × 10^−2^) was used as a quality control sample for the uranium (and protactinium) isotope ratio measurements. The ^237^Np spike was prepared from certified reference material solution from Cetama (CEA, France). The ^237^Np solution was transferred to a Teflon vial and kept under weight control. The solution medium was *cc*HCl/*cc*HNO_3_ to avoid precipitation or adsorption. The ^237^Np concentration was around 900 μg/g, while the *n*(^231^Pa)/*n*(^233^Pa) amount ratio was 0.0464 ± 0.0013. The resin retention studies were performed using a carrier-free ^231^Pa solution (PNP10010) from AEA Technology (UK). The activity concentration was 419 Bq/g.

TK-400 (50–100 μm particle size) extraction chromatographic resin was supplied by Triskem International (Bruz, France). For column preparation 1.8 ml resin was used in a polyethylene Bio-Rad holder (diameter: 6 mm), washed and conditioned with 8 ml *cc*HCl before use. Silica gel (Merck KGaA, 10-40 μm particle size, 0.5 ml bed volume, column diameter: 4 mm) was filled in as slurry in a polyethylene Bio-Rad column holder. The column was washed and conditioned with 3 ml of 4% HNO_3_. Porous Teflon frits (Reichelt Chemietechnik Heidelberg, Germany) were placed gently on the top of the resins to avoid mixing.

### Instrumentation

The Pa, Np and U isotopic analyses were performed using a double-focusing magnetic sector inductively coupled plasma mass spectrometer (ICP-MS) equipped with a single electron multiplier (Element2, Thermo Electron Corp., Bremen, Germany). All measurements were done in low resolution mode (*R* = 300) using a low-flow micro-concentric nebulizer operated in a self-aspirating mode (flow rate was approximately 50 μL/min) in combination with a quartz Stable Introduction System. Concentrations of isotopes of interest were determined as a function of ^231^Pa/^233^Pa and ^233^U/^235^U ratios according to the isotope dilution method (ID-MS). The measured amount contents of ^231^Pa and ^235^U were used to calculate the model ages according to Eq. (1). The measured isotope ratios obtained by ICP-MS were corrected for instrumental mass bias using linear correction [11]. The ^237^Np spike concentration was determined by ICP-MS using external calibration and Bi as an internal standard. The U concentrations and isotopic compositions were also measured by thermal ionization mass spectrometry (TIMS) using a Triton instrument (Thermo Scientific, Bremen, Germany) for U isotopics to confirm the ICP-MS results, but they were not used for the evaluation.

Optimization of the Pa and U separation was monitored by high resolution gamma spectrometry (HRGS) using a well-type HPGe detector (GCW 2022 model, Canberra Industries Inc., USA) with approximately 20% relative efficiency and a resolution of < 1.7 keV at 185.6 keV. The gamma counting system consisted of a Canberra model 2022 amplifier and a Canberra model 8075 analog-to-digital converter. The measured spectra were evaluated using Genie 2000 v2.1 software. The measurement times varied between 600 and 5200 s. All gamma spectrometric measurements were performed at fixed geometries (i.e. relative measurements to the original starting material before the separation). The 49.55 keV gamma peak of ^238^U (emission probability of 0.064%) and 27.4 keV gamma peak of ^231^Pa (emission probability of 11.1%) were used. Background was measured every day.

In order to measure the ^237^Np content and Pa/Np ratio, a sample containing 10 ml of 1 mg/ml ^237^Np stock solution was measured by an extended range HPGe detector with 50% relative efficiency and a resolution of < 1.9 keV at 1.3 MeV using a LabSOCS™ (Laboratory Sourceless Calibration Software) mathematical efficiency calibration software. Spectra were collected for 60,000 s with a DSA-1000 Digital Spectrum Analyzer and evaluated using Genie 2000 v.3.2.1 software. Efficiency calibration was calculated with LabSOCS™. ^237^Np activity was evaluated from the gamma lines at 143.3 and 151.4 keV; for ^233^Pa the lines at 300.3, 312.2, 340.8, 398.6 and 415.8 keV were used.

### Investigated U samples

For the optimization of the Pa/U separation U_3_O_8_ with natural isotopic composition was used [12]. This sample was available in higher quantity and purity. The analysed U reference materials were CRM 125-A (approx. 4% enriched UO_2_ pellet from New Brunswick Laboratory, USA), IRMM-1000b (approx. 3.6% enriched uranium nitrate from EC Joint Research Centre, Geel, Belgium), U100 (approx. 10% enriched U_3_O_8_ from New Brunswick Laboratory, USA) and U630 (approx. 63% enriched U_3_O_8_ from New Brunswick Laboratory, USA). The materials have either a certified production date through the ^230^Th/^234^U model age (CRM 125-A, IRMM-1000b, U630) or known (archive) production date (U100). 60-80 mg of each of the U materials was dissolved in 8 M HNO_3_/0.02 M HF on a hot plate at 80 °C for 24 h resulting about 20 mg U/ml solution.

### Optimization of Pa separation from U matrix

**Fig. 1:**
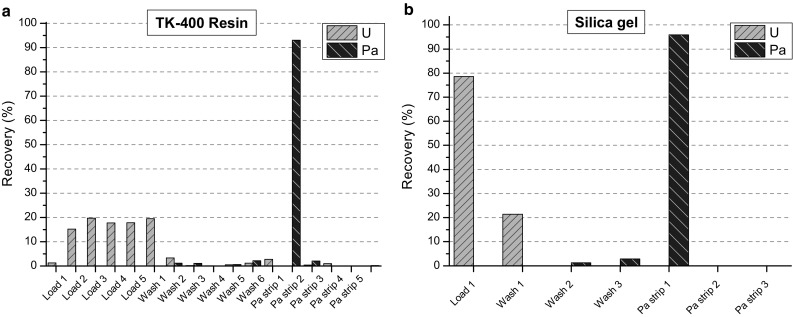
Elution profile of U and Pa using different resins

Two methods were considered for Pa separation: TK-400 extraction chromatography resin [13] and silica gel [8, 10]. Other options, like ion exchange chromatography or extraction chromatography with other resins were excluded, either because of the low Pa recovery or ineffective separation from Np and U [14]. The two methods were tested using natural U_3_O_8_ and ^231^Pa tracer and the measurements were performed by gamma spectrometry. The fractions from the successive elution were collected in 1-ml portions to calculate the U separation factor (defined as the quotient of the Pa/U ratio in the initial material and after the chemical separation) and Pa recovery. HF was removed by adding 20 μl of saturated H_3_BO_3_ and a few drops of HClO_4_. Before loading the solutions in the columns, they were converted to *cc*HCl (TK-400) or 4% HNO_3_ (silica gel). The final volume of the test sample for loading was 5 ml (TK-400) or 1 ml (silica gel), with the U amount of 1.5 mg for both cases. For TK-400 test the wash was 6 × 1 ml of *cc*HCl, and the Pa strip (elution) was completed using 6 × 1 ml of 1 M HCl. For silica gel, as the capacity of the resin is much higher due to the small particle size, after wash with 3 × 1 ml of 4% HNO_3_, Pa was eluted with 3 × 1 ml of 4% HNO_3_/0.02 M HF. The elution profiles of the two resins are shown in Fig. 1.

For TK-400 multiple wash steps were necessary to reduce the U amount in the sample. Collecting the first two Pa strips, a U/Pa separation factor of 1300 could be achieved with a Pa recovery of 95%. When using the silica gel, much higher separation of U could be achieved (separation factor was 1.0 × 10^6^) with a recovery of 96% using only 1 ml strip solution. As has been shown by other studies, Np behaves similarly to U in the separation scheme [8, 10].

Due to the much higher separation factor, the one-step silica gel column was chosen for the following separations. A rapid separation is important for the short-lived ^233^Pa. Another advantage is that use of *cc*HCl can be omitted, which can cause corrosion in the glove-box. Possible clogging of the sample introduction system during the ICP-MS measurement due to the SiO_2_ particles from the silica gel resin (which was the major concern) was avoided by adding 0.4 ml of 4% HNO_3_/0.02 M HF to the eluted Pa fraction.

### ^231^Pa/^235^U ratio measurement of the U samples

Aliquots of the dissolved U CRM samples were transferred gravimetrically to Teflon containers. About 20 mg U was used for each measurement. 300 μl of ^237^Np spike was added gravimetrically to the samples corresponding to about 270 μg ^237^Np and 10 pg ^233^Pa. 25 μl of saturated H_3_BO_3_ and 30 μl of HClO_4_ were added to the samples to remove the HF. The samples were evaporated to almost complete dryness. 200 μl *cc*HNO_3_ was added to the samples together with 25 μl H_3_BO_3_ and 30 μl of HClO_4_. The samples were evaporated again. Overall, this step was repeated three times. Then the samples were taken up to 1 ml of 4% HNO_3_. The samples were loaded on the pre-conditioned silica gel column. After loading the samples, the columns were washed with 4 × 1 ml 4% HNO_3_, and Pa was eluted with 2 × 0.8 ml 4% HNO_3_/0.02 M HF in a polyethylene vial. The ICP-MS measurement was performed soon after the final wash step and Pa elution in order to minimize the ^233^U ingrowth from the ^233^Pa decay. Times of loading, elution and measurement were recorded (see Supplementary Information). Due to the short half-life of ^233^Pa (decay of ^233^Pa is about 2.5% in 24 h) the time span between the separation and measurement has to be recorded and taken into account. The time span was defined as the difference of the sample loading and the ICP-MS measurement. As this span is difficult to measure exactly, a 0.5 h uncertainty was assigned to the length of separation. All samples were measured in duplicate referring to #1 and #2.

### Data evaluation

**Fig. 2:**
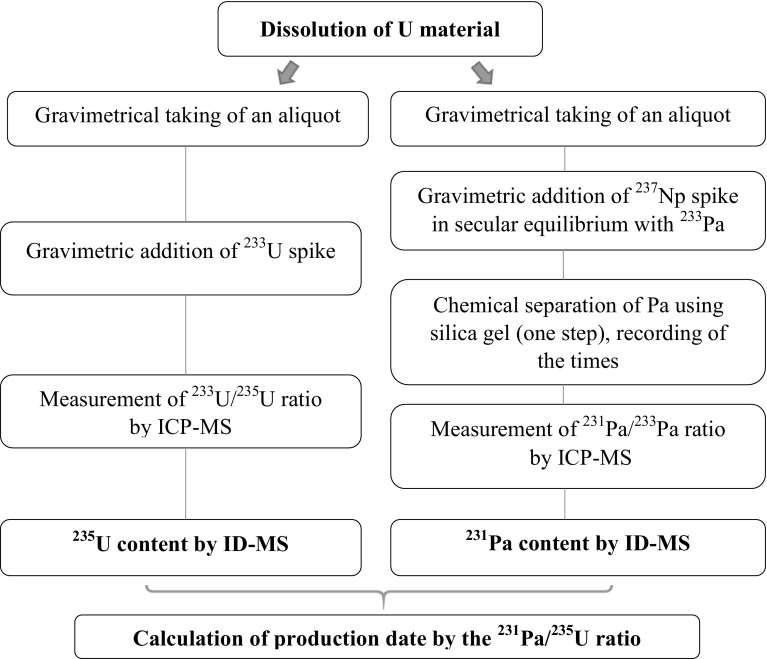
A schematic of the age determination from the ^231^Pa/^235^U ratio

For the age calculations nuclear data from the Decay Data Evaluation Project were used [15]. The ^231^Pa and ^235^U half-lives are 32,670 ± 260 years and 704 ± 1 × 10^6^ years (*k* = 1), respectively. The overall uncertainties were calculated according to ISO/BIPM guide and taking into account the uncertainty of the weight measurements, spike concentrations, measured isotope ratios, nuclear data and elapsed time between the separation and measurement [16]. The given uncertainties are expanded uncertainties with a coverage factor of *k* = 2 if not indicated otherwise. The Pa chemical recovery and U separation factor calculations were carried out by Excel^®^, while for the age calculations commercially available software, GUM Workbench was used [17]. The schematic of the age measurement is shown in Fig. 2.

## Results and discussion

### ^237^Np spike measurement

**Fig. 3:**
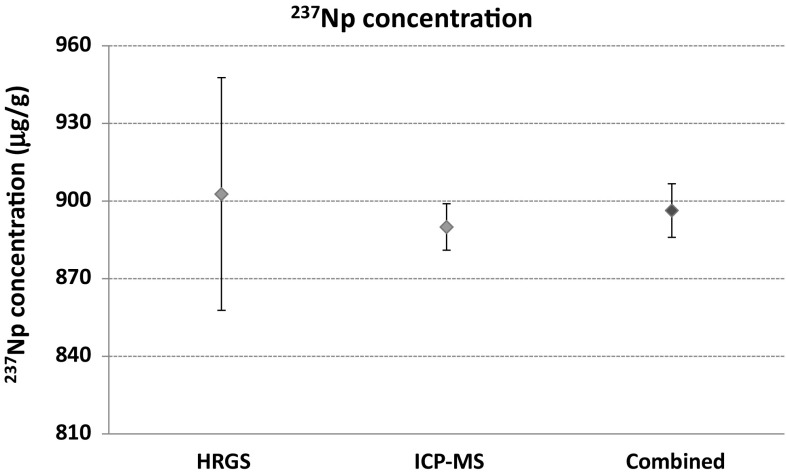
Measurement results of the ^237^Np spike concentration

The ^237^Np spike, which is in secular equilibrium with ^233^Pa, was measured by HRGS and by ICP-MS for the ^237^Np concentration. The HRGS and ICP-MS resulted in 903 ± 45 μg/g (*k *= 1) and 890 ± 9 μg/g (*k *= 1), respectively. The determined values based on different principles (activity vs. mass) resulted in comparable results (Fig. 3). A combined value was obtained from the different results by taking the mean weighted by the inverse of the variances [15]. The combined value of 896 ± 10 μg/g (*k *= 1) was used for the chronometric studies.

It has to be noted that ^233^Pa amount can also be measured with gamma spectrometry, not only the ^237^Np amount in the sample. However, in order to compare the ^237^Np values obtained by HRGS and ICP-MS result the ^233^Pa result was not used. With gamma spectrometry the possible adsorption of Pa on the inner surface of the vial was checked: the Pa/Np ratio was 1.00 ± 0.03 with the standing vial, while turning it upside down the ratio was 0.96 ± 0.06. If Pa would adsorb on the surface of the vial, the Pa/Np ratio would have been much lower in the latter case as the Pa efficiency is lower due to the higher distance from the detector. It was concluded that Pa was in the liquid phase together with Np, and no fractionation had occurred between the radionuclides, i.e. no adsorption had taken place on the vial surfaces.

### ^231^Pa/^235^U model production date results

**Fig. 4:**
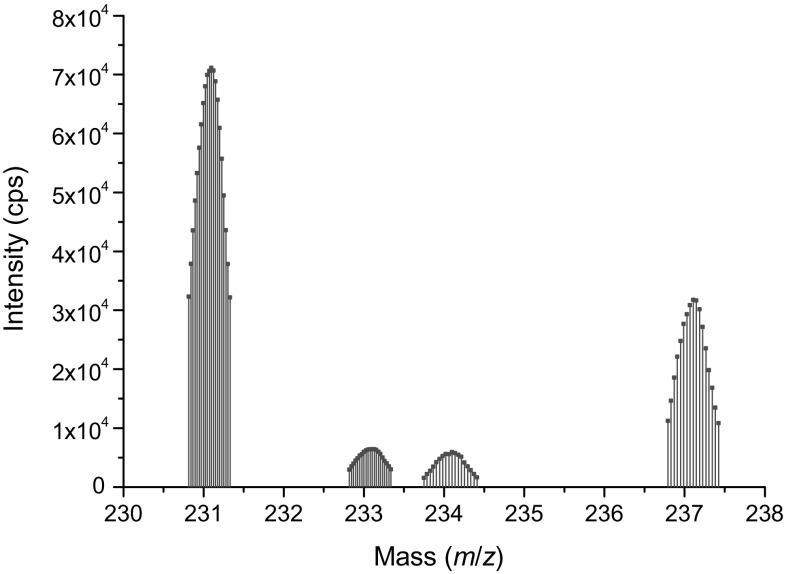
Typical ICP-MS spectrum (U100 #1). The shown mass regions are the selected masses for measurement (*m*/*z* of ^231^Pa, ^233^Pa, ^234^U and ^237^Np). Major U isotopes were not measured due to the possible high signal

A typical ICP-MS spectrum is shown in Fig. 4. Even though there was only a single separation, U and Np were well separated from the Pa fraction. The ^231^Pa/^235^U mass ratios together with the ^231^Pa/^235^U model ages and production dates are summarized in Table 1. The measured ^231^Pa and ^235^U concentrations used for the model age calculation are collected in the Supplementary Information. The ^235^U values measured by isotope dilution TIMS were used to check the ICP-MS results, but they were not used for the model age calculation.

**Table 1:** Measured ^231^Pa/^235^U mass ratios and production dates

Sample	^231^Pa/^235^U mass ratio	Standard uncertainty	^231^Pa/^235^U model age (year)	Expanded uncertainty (year, *k *= 2)	Measured ^231^Pa/^235^U model production date
CRM 125-A #1	2.278 × 10^−8^	4.2 × 10^−10^	23.54	0.88	26 December 1994
CRM 125-A #2	2.269 × 10^−8^	3.2 × 10^−10^	23.45	0.67	27 January 1995
IRMM1000 #1	5.926 × 10^−9^	1.2 × 10^−10^	6.12	0.24	27 May 2012
IRMM1000 #1	6.006 × 10^−9^	1.0 × 10^−10^	6.21	0.21	24 April 2012
U100 #1	5.619 × 10^−8^	7.5 × 10^−10^	58.1	1.6	03 June 1960
U100 #2	5.813 × 10^−8^	9.6 × 10^−10^	60.1	2.0	04 June 1958
U630 #1	2.815 × 10^−8^	4.0 × 10^−10^	29.09	0.83	07 June 1989
U630 #2	2.805 × 10^−8^	3.8 × 10^−10^	28.99	0.79	14 July 1989

The replicate results agree well with each other. The age values have a relative expanded uncertainty of 2.7–3.9%, which is determined mainly by the low abundant ^231^Pa measurement: about 90–95% of the total uncertainty contribution derives from the ^231^Pa analysis. Out of the ^231^Pa measurement uncertainty the major components are ^231^Pa/^233^Pa ratio measurements in the blend by ICP-MS and ^237^Np (^233^Pa) spike concentration. Other parameters, like weight measurement, nuclear data, length of separations or other ratio measurements, correspond to less than 10% of the to the total uncertainty.

**Fig. 5:**
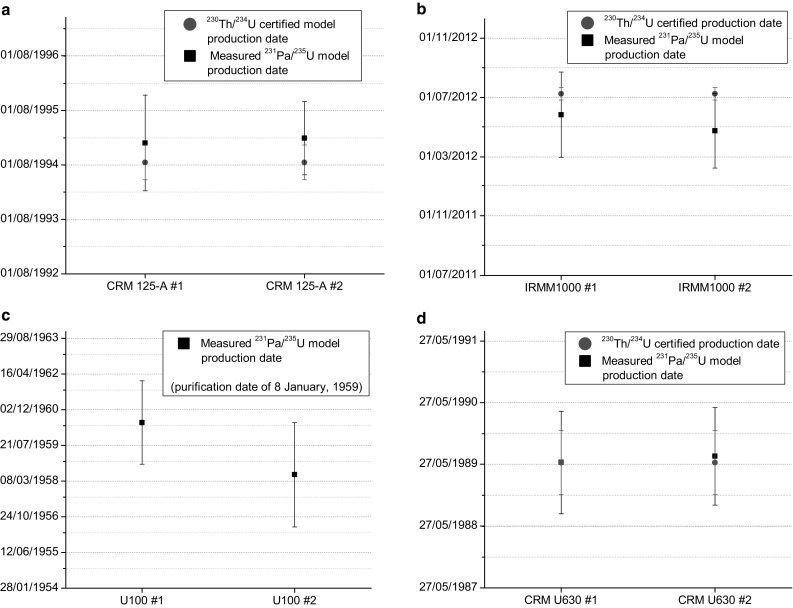
^231^Pa/^235^U model production date of the measured U samples. In **a**, **b**, **d** the certified production dates by the ^230^Th/^234^U ratio are also given

The measured values agree well with the reported values [8]. For three materials, CRM 125-A, IRMM-1000 and U630, the measured ^231^Pa/^235^U model ages are shown together with the certified production dates (Fig. 5). As the obtained ^231^Pa/^235^U ages agree with the ages obtained from the ^230^Th/^234^U ratio, it indicates that at the time of production of these materials Th and Pa had been quantitatively removed from uranium. For IRMM-1000 both obtained values are slightly lower than the certified age. Although this is not significant, it was also observed during the REIMEP-22 inter-laboratory comparison [18]. For U100 the measured ^231^Pa/^235^U production dates are in agreement with the purification date of 8 January, 1959 [8].

## Conclusions

An improved method was developed for the chemical separation of Pa and determination of the production date of uranium materials based on the ^231^Pa/^235^U ratio measurement. The ^233^Pa spike necessary for the quantification of the ^231^Pa concentration was obtained by adding ^237^Np solution directly to the sample, where the ^233^Pa was in secular equilibrium with its parent nuclide. Therefore, there is no need to separate beforehand ^233^Pa from ^237^Np, and the spike is continuously available without the need of milking. Omitting the milking step the possibility of Pa loss through adsorption is reduced significantly. The possible fractionation between Np and Pa as well as the potential adsorption of the spike was checked by gamma spectrometry. The proposed one-step separation and the measurement method are faster and lower uncertainty can be achieved than with alpha spectrometry and it is applicable even for young low-enriched uranium samples. The method can be applied for uranium found out of regulatory control (i.e. in nuclear security) and for safeguards samples to complement the ^230^Th/^234^U model age, i.e. to check if concurrent ages are obtained.

## Electronic supplementary material

Below is the link to the electronic supplementary material.

Supplementary material 1 (DOC 73 kb)
