# Supplementary material for: Measurement of the 231Pa/235U ratio for the age determination of uranium materials
Source: J Radioanal Nucl Chem. 2018 Nov 11;318(3):1565–71. doi: 10.1007/s10967-018-6247-9 (PMC6267122; doi:10.1007/s10967-018-6247-9)
Supplement: Supplementary file 1 — Supplementary material 1 (DOC 73 kb) [file 10967_2018_6247_MOESM1_ESM.doc]

Supplementary information

**Table 1** Separation times

| **Sample** | **Loading** | **Elution** | **Measurement** | **Length in hour*** |
| --- | --- | --- | --- | --- |
| IRMM-1000 #1 | 10/July/2018 14:00 | 11/July/2018 09:15 | 11/July/2018 11:15 | **21.25** |
| IRMM-1000 #2 | 10/July/2018 14:00 | 11/July/2018 09:15 | 11/July/2018 13:08 | **23.13** |
| CRM 125-A #1 | 10/July/2018 14:00 | 11/July/2018 09:15 | 11/July/2018 11:35 | **21.58** |
| CRM 125-A #2 | 10/July/2018 14:00 | 11/July/2018 09:15 | 11/July/2018 13:47 | **23.78** |
| U100 #1 | 10/July/2018 14:00 | 11/July/2018 09:15 | 11/July/2018 11:55 | **21.92** |
| U100 #2 | 10/July/2018 14:00 | 11/July/2018 09:15 | 11/July/2018 14:07 | **24.12** |
| U630 #1 | 10/July/2018 14:00 | 11/July/2018 09:15 | 11/July/2018 12:16 | **22.27** |
| U630 #2 | 10/July/2018 14:00 | 11/July/2018 09:15 | 11/July/2018 14:25 | **24.42** |

* Length: defined as the measurement time minus loading. 0.5 h was assigned to the time length uncertainty.

**Table 2** ICP-MS ^231^Pa amount content (Ref. date: 11/July/2018)

| **Sample** | **^231^Pa concentration (ng/g)** | **Uncertainty (ng/g, *k* =2)** |
| --- | --- | --- |
| IRMM-1000 #1 | 3.89 × 10^-3^ | 0.15 × 10^-3^ |
| IRMM-1000 #2 | 3.92 × 10^-3^ | 0.13 × 10^-3^ |
| CRM 125-A #1 | 0.01596 | 0.00059 |
| CRM 125-A #2 | 0.01595 | 0.00045 |
| U100 #1 | 0.1220 | 0.0032 |
| U100 #2 | 0.1259 | 0.0041 |
| U630 #1 | 0.3232 | 0.0090 |
| U630 #2 | 0.3236 | 0.0086 |

**Table 3** ICP-MS and TIMS ^235^U results

| **Sample** | **ICP-MS results** | | **TIMS results*** | |
| --- | --- | --- | --- | --- |
|  | **^235^U concentration (mg/g)** | **Uncertainty (mg, *k* =2)** | **^235^U concentration (mg/g)** | **Uncertainty (mg, *k* =2)** |
| IRMM-1000 #1 | 0.6564 | 0.0026 | 0.6591 | 0.0012 |
| IRMM-1000 #2 | 0.6564 | 0.0029 | 0.6587 | 0.0012 |
| CRM 125-A #1 | 0.7006 | 0.0028 | 0.7039 | 0.0013 |
| CRM 125-A #2 | 0.7030 | 0.0029 | 0.7038 | 0.0013 |
| U100 #1 | 2.171 | 0.012 | 2.1749 | 0.0072 |
| U100 #2 | 2.166 | 0.012 | 2.1712 | 0.0072 |
| U630 #1 | 11.482 | 0.059 | 11.480 | 0.038 |
| U630 #2 | 11.537 | 0.056 | 11.483 | 0.038 |

* Only the ICP-MS values were used for the ^231^Pa/^235^U model age determination
